# Prognostic impact of secondary versus de novo ontogeny in acute myeloid leukemia is accounted for by the European LeukemiaNet 2022 risk classification

**DOI:** 10.1038/s41375-023-01985-y

**Published:** 2023-07-31

**Authors:** Michael J. Hochman, Megan Othus, Robert P. Hasserjian, Alex Ambinder, Andrew Brunner, Mary-Elizabeth M. Percival, Christopher S. Hourigan, Ronan Swords, Amy E. DeZern, Elihu H. Estey, Judith E. Karp

**Affiliations:** 1 Division of Hematological Malignancies and Bone Marrow Transplantation, Sidney Kimmel Comprehensive Cancer Center, Johns Hopkins University, Baltimore, MD USA; 2 Public Health Sciences Division, Fred Hutchinson Cancer Center, Seattle, WA USA; 3 Department of Pathology, Massachusetts General Hospital, Boston, MA USA; 4 Leukemia Program, Massachusetts General Hospital Cancer Center, Boston, MA USA; 5 Department of Medicine, Division of Hematology, University of Washington, Seattle, WA USA; 6 Clinical Research Division, Fred Hutchinson Cancer Center, Seattle, WA USA; 7 National Heart, Lung, and Blood Institute, National Institutes of Health, Bethesda, MD USA; 8 Division of Hematology/Medical Oncology, Oregon Health & Science University, Portland, OR USA

**Keywords:** Acute myeloid leukaemia, Acute myeloid leukaemia, Risk factors, Cancer genomics

## Abstract

Secondary AML (sAML), defined by either history of antecedent hematologic disease (AHD) or prior genotoxic therapy (tAML), is classically regarded as having worse prognosis than de novo disease (dnAML). Clinicians may infer a new AML diagnosis is secondary based on a history of antecedent blood count (ABC) abnormalities in the absence of known prior AHD, but whether abnormal ABCs are associated with worse outcomes is unclear. Secondary-type mutations have recently been incorporated into the European LeukemiaNet (ELN) 2022 guidelines as adverse-risk features, raising the question of whether clinical descriptors of ontogeny (i.e., de novo or secondary) are prognostically significant when accounting for genetic risk by ELN 2022. In a large multicenter cohort of patients (*n* = 734), we found that abnormal ABCs are not independently prognostic after adjusting for genetic characteristics in dnAML patients. Furthermore, history of AHD and tAML do not confer increased risk of death compared to dnAML on multivariate analysis, suggesting the prognostic impact of ontogeny is accounted for by disease genetics as stratified by ELN 2022 risk and *TP53* mutation status. These findings emphasize the importance that disease genetics should play in risk stratification and clinical trial eligibility in AML.

## To the Editor:

Classification of acute myeloid leukemia (AML) by ontogeny distinguishes patients with known antecedent hematologic disease (AHD) or prior chemoradiation exposure (therapy-related, tAML) from those patients without these etiologic features (de novo, dnAML). Large population-based studies support an association between both AML post-AHD and tAML (collectively known as secondary AML, sAML) and worse prognosis compared to dnAML, especially in younger patients [1, 2]. Next generation sequencing (NGS) technologies have allowed for improved characterization of AML genetics; specific genetic alterations are now associated with ontogeny-related groups [3]. Additionally, tAML with favorable-risk genetics appears to have transcriptomic features and clinical outcomes similar to genetically favorable-risk dnAML [4, 5]. Similarly, although *TP53*-mutated AML often results from cytotoxic therapy exposure, *TP53*-mutated disease has dismal outcomes regardless of prior therapy history [6]. Altogether, these studies suggest disease genetics may drive the outcomes classically attributed to ontogeny.

While ontogeny provides a convenient means to readily risk stratify AML patients at diagnosis, it also has been frequently used to determine eligibility for clinical trials. However, patients with a new AML diagnosis may have not been formally diagnosed with AHD due to patient refusal to undergo bone marrow examination, lack of access to care, or prior non-diagnostic bone marrow samples. Alternatively, patients may be inferred to have sAML from AHD (“post-AHD sAML”) due to a history of antecedent blood count (ABC) abnormalities, though such abnormalities may be multifactorial and unrelated to a primary marrow disorder. Whether such patients with abnormal ABCs but without pathologically proven AHD should be considered equivalent to sAML is unclear. Furthermore, the poor prognosis of sAML may reflect the adverse-risk genetics that often accompanies a secondary etiology as opposed to the historical background itself. Importantly, dnAML cases carrying secondary-type mutations [3] (e.g., *ASXL1*, *BCOR*, *EZH2*, etc.) or cytogenetic aberrations have poor clinical outcomes [7]. Accordingly, the 2022 European LeukemiaNet (ELN) risk classification [8] incorporated secondary-type mutations in its adverse-risk group. Herein, we evaluated whether ABC abnormalities, documented AHD, or exposure to prior cytotoxic therapy were independently prognostic after accounting for ELN 2022 risk category and *TP53* mutation at time of AML presentation.

Clinical, histopathologic, cytogenetic, and molecular genetic data were collected on 734 adults with newly diagnosed AML (as defined by the 5^th^ edition of the WHO Classification of myeloid neoplasms [9]) from Johns Hopkins Hospital, Baltimore, MD (*n* **=** 388) and Massachusetts General Hospital, Boston, MA (*n* **=** 346). Post-AHD sAML cases were defined by prior confirmed pathologic diagnosis of myelodysplastic syndrome (MDS) or myelodysplastic/myeloproliferative neoplasm (MDS/MPN; corresponding to AML-myelodysplasia-related [AML-MR] in the 5th edition WHO Classification [9]) or a myeloproliferative neoplasm (MPN; corresponding to MPN in blast phase [9]). Patients with dnAML were evaluated for abnormal ABCs at least 28 days and up to 2 years prior to the AML diagnosis. Univariate and multivariable Cox models were used to evaluate associations between OS and RFS and relevant covariates. Additional information on our methods can be found in our Supplementary Materials.

Of the 734 patients in the cohort, 488 (66%) had dnAML, 123 (17%) had post-AHD AML, and 123 (17%) had tAML (Supplementary Table 1). Of the dnAML patients, 88 had normal and 162 had abnormal ABCs documented; ABCs were not available in the remaining 238 patients. Patients with post-AHD sAML and tAML patients were, on average, older than patients with dnAML (median age 71 and 67 versus 63 years respectively, *P* < 0.001) and had a greater proportion with poor performance status (28% and 29% versus 18% respectively, *P* = 0.006). AHD was diagnosed a median of 461 days prior to post-AHD sAML (range 8–5 959 days). The majority of AHD cases were MDS (Supplementary Table 2). A greater proportion of patients with post-AHD sAML and tAML had adverse ELN risk compared to patients with dnAML (69% and 82% versus 53% respectively, *P* < 0.001) and a greater proportion were *TP53*-mutated (36% and 25% versus 12% respectively, *P* < 0.001). A smaller proportion of patients with post-AHD sAML and tAML received high/intermediate-intensity treatment versus dnAML (34% and 43% versus 66% respectively, *P* < 0.001).

**Fig. 1:**
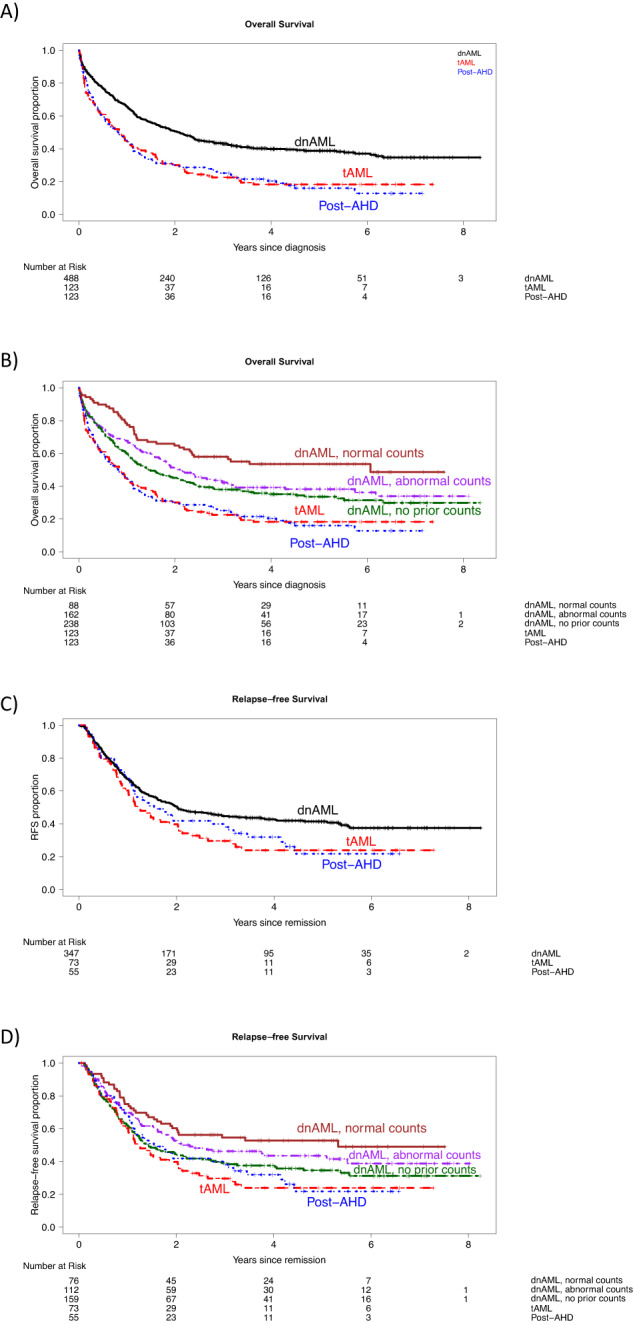
Kaplan-Meier estimates of overall and relapse free survival stratified by ontogeny and antecedent blood count abnormalities. **A** Overall survival stratified by ontogeny: de novo acute myeloid leukemia (dnAML), secondary AML post-antecedent hematologic disease (AHD), and therapy-related AML (tAML). **B** Overall survival stratified by ontogeny and antecedent blood count abnormalities in dnAML. **C** Relapse free survival stratified by ontogeny. **D** Relapse free survival stratified by ontogeny and antecedent blood count abnormalities in dnAML.

Patients with dnAML had significantly longer survival than those with either post-AHD sAML or tAML (Fig. 1A), and survival differed among patients with dnAML based on their history of ABC abnormalities (Fig. 1B). Relapse-free survival in these subgroups followed similar patterns (Fig. 1C, D). On multivariable analysis (Supplementary Table 3), however, there was no significant difference in hazard of death between those with abnormal versus normal ABCs (hazard ratio [HR] 1.22, 95% confidence interval [CI] 0.84-1.77).

Given that abnormal ABCs were not independently prognostic in dnAML on multivariable analysis, we grouped all dnAML cases together on subsequent analyses regardless of ABCs. Among patients with ELN adverse-risk disease, a higher proportion of post-AHD sAML patients had secondary-type mutations compared to patients with dnAML or tAML (58% versus 40% and 24%, respectively, *P* < 0.001). Two hundred fifty-six of the 488 patients with dnAML (52%) received HCT; 42/123 (34%) and 44/123 (36%) received HCT for post-AHD sAML and tAML, respectively. In a multivariable model, patients with post-AHD sAML and tAML did not have a significantly increased hazard of death relative to those with dnAML (HR 1.12, 95% CI 0.88–1.42 and 1.21, 95% CI 0.95–1.53, respectively; see Table 1). ELN risk and *TP53* mutation status remained independently significant in the multivariable model.

**Table 1:** Cox regression models for overall survival. Baseline hazard was stratified by institution (Hopkins versus MGH).

	Univariate HR (95% CI)	Multivariable HR (95% CI)
AML Group (ref = dnAML)		
Post-AHD sAML	1.81 (1.44–2.27)	1.12 (0.88–1.42)
tAML	1.77 (1.41–2.23)	1.21 (0.95–1.53)
Age at diagnosis (per 10 years)	1.30 (1.21–1.39)	1.11 (1.03–1.20)
Poor PS (ref = Good PS)	3.10 (2.51–3.82)	2.39 (1.91–2.98)
ELN22 Group (ref = ELN22 favorable)		
ELN22 intermediate	1.52 (1.07–2.17)	1.90 (1.32–2.73)
ELN22 adverse	3.05 (2.30–4.05)	2.71 (2.00–3.68)
*TP53* mutant (ref = *TP53* wild-type)	3.15 (2.55–3.89)	1.74 (1.38–2.20)
Low-intensity chemotherapy (ref = high/intermediate-intensity chemotherapy)	2.37 (1.98–2.83)	1.23 (0.98–1.56)
Transplant (ref=no transplant)	0.38 (0.31–0.47)	0.45 (0.36–0.56)

Transplant was analyzed as a time-dependent covariate.

Missing PS, missing *TP53*, and ELN unknown were analyzed as separate categories (to not exclude patients missing those data from the multivariable model; results not printed for brevity).

*ref* reference, *HR* hazard ratio, *CI* confidence interval, *sAML* secondary acute myeloid leukemia, *AHD* antecedent hematologic disease, *dnAML* de novo AML, *tAML* therapy-related AML, *PS* performance status, *ELN22* European LeukemiaNet 2022.

Our analysis had several key findings. First, we found that abnormal ABCs do not independently impact outcomes in dnAML patients, highlighting that secondary ontogeny cannot reliably be deduced from clinical history of abnormal ABCs in the absence of a formal AHD diagnosis. Antecedent thrombocytopenia was previously found to be independently prognostic in a smaller cohort (*n* = 140) of dnAML patients [10]; however, this study did not adjust for gene mutations as ours did. Notably, patients without recorded ABCs maintained independently worse prognosis on multivariable analysis (HR 1.94, 95% CI 1.36–2.76). We recognize that our approach to evaluating for ABC abnormalities is limited by the lack of clinical context that clinicians often use when evaluating such a history, but our objective methodology is reproducible and avoids bias inherent to determining which count abnormalities may suggest an undiagnosed AHD.

Next, we found that the prognostic impact of disease-related genetic risk, as measured by ELN 2022 and *TP53* mutation status, supersedes the risk conferred by AML ontogeny. This finding emphasizes the role that disease genetics should play in determining patient prognosis and driving therapeutic decision making. While the turnaround time of cytogenetics and NGS assays has posed challenges in using genetics to influence up-front treatment choice, the BEAT AML Master trial demonstrated that a precision medicine approach is theoretically possible in the subset of newly diagnosed AML patients who are able to wait a 7-day period prior to starting therapy [11]. Moreover, the approval of molecularly-targeted therapies in the front-line for AML (such as midostaurin and ivosidenib for *FLT3*-ITD and *IDH1*-mutated disease, respectively) illustrates the utility of genetics in the pre-treatment setting and may help drive more rapid turnaround of genetic testing to inform treatment choice [12]. Ontogeny will always remain valuable to leukemia specialists by providing important context to AML presentation, hence its inclusion as a disease qualifier in classification guidelines [8, 9]. However, our findings suggest that AML clinical trials should enroll patients from genetically-defined groups as opposed to more genetically heterogenous groups defined by ontogeny, in order to most definitively determine who is most likely to benefit from an intervention.

One recently published single-center study found ontogeny was independently prognostic even after adjusting for AML genetic risk; the hazard of death for tAML or post-AHD sAML was over two-fold higher than that for dnAML on multivariate analysis [13]. Possible reasons for our differing results include the smaller single institution patient cohort and lack of a surrogate for baseline comorbidities in the McCarter study; ours attempted to capture this via use of baseline performance status. Additionally, interactions between different genomic abnormalities, such as co-mutation patterns or multi-hit *TP53* mutations, may account for some of the disease risk attributed to ontogeny in the McCarter paper; for example, one recent study found that two or more (rather than at least one) secondary-type mutation better defined a high-risk AML cohort [14]. Together our reports suggest that disease genetics ultimately have a greater impact on survival outcomes than ontogeny.

Finally, we found that *TP53* mutation status appears to confer additional independent disease risk even when accounting for ELN adverse status and ontogeny. Given our findings and the poor outcomes known to be associated with this disease group [6] especially in the presence of complex karyotype [15], we suggest that AML patients with *TP53*-mutated disease be considered to represent a “very adverse” group separate from the ELN 2022 adverse-risk group.

In summary, ABCs did not provide independent prognostic information for dnAML patients after accounting for ELN 2022 genetic risk and *TP53* mutation status. Additionally, neither post-AHD sAML nor tAML maintained prognostic associations with shorter OS in the multivariable model. These findings suggest that comprehensive genetic categorization, as opposed to clinical descriptors of ontogeny, should play a primary role in clinical risk stratification and trial eligibility in newly diagnosed AML.

## Supplementary information

Supplemental Materials

Supplementary Table 1

Supplementary Table 2

Supplementary Table 3

## Data Availability

Please contact the corresponding author for data requests; data will be shared per each institution’s policies and procedures.

## Code availability

The computer code used in R to generate the results in this paper is available for access by emailing Dr. Megan Othus at mothus@fredhutch.org.
