# Supplementary material for: Prognostic impact of secondary versus de novo ontogeny in acute myeloid leukemia is accounted for by the European LeukemiaNet 2022 risk classification
Source: Leukemia. 2023 Jul 31;37(9):1915–8. doi: 10.1038/s41375-023-01985-y (PMC10457181; doi:10.1038/s41375-023-01985-y)
Supplement: Supplementary file 1 — Supplemental Materials [file 41375_2023_1985_MOESM1_ESM.docx]

**Supplementary Materials**

**Methods**

As MPN may be diagnosed presumptively based on thrombocytosis or polycythemia and presence of a *JAK2*, *CALR*, or *MPL* mutation, these cases were considered equivalent to a prior AHD even if an antecedent bone marrow examination was not performed. Cases with antecedent aplastic anemia or paroxysmal nocturnal hemoglobinuria were also considered as post-AHD sAML. tAML cases were defined by history of prior cytotoxic chemotherapy, radiation, or radioisotope treatment. tAML cases either had 20% blasts or greater at diagnosis, extramedullary disease, or an AML-defining genetic alteration with less than 20% blasts as defined in the 5^th^ edition of the WHO myeloid disease classification (Khoury et al. *Leukemia* 2022). Cases in which patients received prior cytotoxic therapies and developed a therapy-related myeloid neoplasm (including MDS or MDS/MPN) prior to AML diagnosis were classified as tAML (n=27).

Abnormal antecedent blood counts were defined by those values were outside the internal reference range for any of the following lab values: white blood cell count, hemoglobin level, platelet count, and absolute neutrophil, monocyte, or eosinophil counts. Additionally, patients with documented clinical history of cytopenias or cytoses were considered as having previously abnormal blood counts. Patients were classified as “ELN 2022 unknown” if no NGS panel was collected 21 days prior to or greater than 7 days after AML diagnosis, or if insufficient genetic testing was done to confirm the ELN risk group (n=93).

AML therapies were classified either as high/intermediate-intensity (e.g., 7+3 or high-dose cytarabine) or low-intensity (e.g., hypomethylating agent/venetoclax therapy or supportive care). Complete remission (CR) was defined per standard response criteria (Döhner H, Wei AH, Appelbaum FR, et al. *Blood* 2022). Overall survival (OS) was measured from first date of AML-directed therapy except for patients receiving supportive care, in which case it was measured from AML diagnosis date. Relapse-free survival (RFS) was measured from the date of first CR to the first of relapse or death from any cause. Patients last known to be alive were censored at date of last contact for OS; those last known to be in CR were censored for RFS. Wilcoxon rank-sum and Fisher’s exact tests were used to compare groups. The following covariates were evaluated in our Cox models (quantitative unless otherwise noted): age, performance status (Eastern Cooperative Oncology [ECOG] group 0-1 as good versus 2-4 as poor), therapy intensity, ELN 2022 risk, TP53 mutation, prior abnormal counts, and hematopoietic stem cell transplant (HCT) status (evaluated as a time-dependent covariate). Analyses were performed using R version 4.3.0. The study protocol was approved by Johns Hopkins School of Medicine and the Massachusetts General Hospital IRB committees. Informed consent was not obtained from study subjects given the study’s retrospective nature; the IRB committees waived HIPPA Privacy Authorization given the project involved no more than minimal risk to the individual participants.

**Supplementary Table and Figure Legends**

**Supplementary Table 1:** Demographic and clinical characteristics of patient population as grouped by ontogeny and antecedent blood count abnormalities (for de novo disease). Median (range) or N (%) reported.

dnAML = de novo acute myeloid leukemia; sAML = secondary AML; AHD = antecedent hematologic disease; tAML = therapy-related AML; PS = performance status; ELN = European LeukemiaNet; Int=Intermediate.

**Supplementary Table 2:** Type of antecedent hematologic disease (AHD) for patients diagnosed with sAML. N (%) reported.

MDS = myelodysplastic syndromes; MDS/MPN = myelodyplastic/myeloproliferative overlap neoplasm; Ph- MPN = Philadelphia chromosome negative myeloproliferative neoplasm; CML = chronic myeloid leukemia; AA = aplastic anemia.

**Supplementary Table 3:** Cox regression models for overall survival including ontogeny and antecedent blood count abnormalities. Baseline hazard was stratified by institution (Hopkins versus MGH). Transplant was analyzed as a time-dependent covariate.

ref = reference; HR= hazard ratio; CI = confidence interval; sAML = secondary acute myeloid leukemia; AHD= antecedent hematologic disease; dnAML = de novo AML; tAML = therapy-related AML; PS = performance status; ELN22 = European LeukemiaNet 2022; int = intermediate. Missing PS, missing *TP53*, and ELN 2022 unknown were analyzed as separate categories (to not exclude patients missing those data from the multivariable model; results not printed for brevity).
