# Supplementary material for: Prognostic impact of secondary versus de novo ontogeny in acute myeloid leukemia is accounted for by the European LeukemiaNet 2022 risk classification
Source: Leukemia. 2023 Jul 31;37(9):1915–8. doi: 10.1038/s41375-023-01985-y (PMC10457181; doi:10.1038/s41375-023-01985-y)
Supplement: Supplementary file 2 — Supplementary Table 1 [file 41375_2023_1985_MOESM2_ESM.docx]

| ***Factor*** | ***dnAML by count abnormalities***  ***(n=488)*** | | | ***sAML by type***  ***(n=246)*** | |
| --- | --- | --- | --- | --- | --- |
|  | Normal counts  (n=88) | Abnormal counts  (n=162) | No Recorded Counts  (n=238) | Post-AHD sAML  (n=123) | tAML  (n=123) |
| **Age (years)** | 60.5 (15, 87) | 65 (24, 91) | 62 (15, 90) | 71 (26, 88) | 67 (14, 83) |
| **Sex**  **Female** | 46 (52) | 59 (36) | 99 (42) | 37 (30) | 63 (51) |
| **Performance Status**  **Good PS**  **Poor PS** | 68 (89)  8 (11) | 124 (81)  29 (19) | 172 (81)  41 (19) | 83 (72)  33 (28) | 78 (71)  32 (29) |
| **ELN 2022 Risk**  **Favorable**  **Intermediate**  **Adverse**  **Unknown** | 32 (36)  18 (20)  34 (39)  4 (5) | 38 (23)  27 (17)  79 (49)  18 (11) | 47 (20)  41 (17)  115 (48)  35 (15) | 8 (7)  10 (8)  83 (67)  22 (18) | 15 (12)  19 (15)  76 (62)  13 (11) |
| ***TP53* status**  **Wildtype**  **Mutant**  **Missing** | 68 (77)  13 (15)  7 (8) | 134 (83)  20 (12)  8 (5) | 198 (83)  24 (10)  16 (7) | 88 (72)  30 (24)  5 (4) | 76 (62)  42 (34)  5 (4) |
| **Therapy Type**  **High/Int-Intensity**  **Low-Intensity** | 71 (81)  17 (19) | 95 (59)  67 (41) | 156 (66)  82 (34) | 42 (34)  81 (66) | 53 (43)  70 (57) |
