# Supplementary material for: Prognostic impact of secondary versus de novo ontogeny in acute myeloid leukemia is accounted for by the European LeukemiaNet 2022 risk classification
Source: Leukemia. 2023 Jul 31;37(9):1915–8. doi: 10.1038/s41375-023-01985-y (PMC10457181; doi:10.1038/s41375-023-01985-y)
Supplement: Supplementary file 3 — Supplementary Table 2 [file 41375_2023_1985_MOESM3_ESM.docx]

| **AHD** | **Post-AHD sAML**  **(n=123)** | **tAML with History of AHD**  **(n=27)** |
| --- | --- | --- |
| **MDS** | 80 (65) | 20 (74) |
| **MDS/MPN** | 26 (21) | 2 (7.4) |
| **Ph- MPN** | 12 (9.7) | 3 (11.1) |
| **CML** | 2 (1.6) | 0 (0) |
| **AA** | 1 (0.8) | 0 (0) |
| **Other** | 2 (1.6) | 2 (7.4) |
