# Supplementary material for: Prognostic impact of secondary versus de novo ontogeny in acute myeloid leukemia is accounted for by the European LeukemiaNet 2022 risk classification
Source: Leukemia. 2023 Jul 31;37(9):1915–8. doi: 10.1038/s41375-023-01985-y (PMC10457181; doi:10.1038/s41375-023-01985-y)
Supplement: Supplementary file 4 — Supplementary Table 3 [file 41375_2023_1985_MOESM4_ESM.docx]

|  | *Univariate*  *HR (95% CI)* | *Multivariable*  *HR (95% CI)* |
| --- | --- | --- |
| AML Group (ref = dnAML with prior normal counts) |  |  |
| dnAML with prior abnormal counts | 1.54 (1.07, 2.22) | 1.22 (0.84-1.77) |
| dnAML with no prior recorded counts | 1.77 (1.25, 2.49) | 1.94 (1.36, 2.76) |
| Post-AHD sAML | 2.76 (1.92-3.98) | 1.59 (1.09-2.33) |
| tAML | 2.72 (1.89-3.91) | 1.72 (1.19-2.51) |
| Age at diagnosis (per 10 years) | 1.30 (1.21-1.39) | 1.13 (1.05-1.23) |
| Poor PS (ref = Good PS) | 3.10 (2.51-3.82) | 2.47 (1.98-3.09) |
| ELN22 Group (ref = ELN22 favorable) |  |  |
| ELN22 intermediate | 1.52 (1.07-2.17) | 1.86 (1.29-2.67) |
| ELN22 adverse | 3.05 (1.43-2.94) | 2.66 (1.96-3.61) |
| *TP53* mutant  (ref = *TP53* wild-type) | 3.15 (2.55-3.89) | 1.86 (1.46-2.36) |
| Low-intensity chemotherapy  (ref = high/int-intensity chemotherapy) | 2.37 (1.98 -2.83) | 1.22 (0.97-1.54) |
| Transplant (ref = no transplant) | 0.38 (0.31-0.47) | 0.46 (0.37-0.58) |
